# Supplementary material for: Mycobacterial and Human Ferrous Nitrobindins: Spectroscopic and Reactivity Properties
Source: Int J Mol Sci. 2021 Feb 7;22(4):1674. doi: 10.3390/ijms22041674 (PMC7915275; doi:10.3390/ijms22041674)
Supplement: Supplementary file 1 [file ijms-22-01674-s001.pdf]

Supplementary Material

Mycobacterial and Human Ferrous Nitrobindins: Spectroscopic and Reactivity Properties

Giovanna De Simone [1,†], Alessandra di Masi [1,†], Alessandra Pesce [2], Martino Bolognesi [3,4], Chiara Ciaccio [5], Lorenzo Tognaccini [6], Giulietta Smulevich [6], Stefania Abbruzzetti [7], Cristiano Viappiani [7], Stefano Bruno [8], Sara Della Monaca [9], Donatella Pietraforte [9], Paola Fattibene [9], Massimo Coletta [5,*] and Paolo Ascenzi [10,*]

1 Dipartimento di Scienze, Università degli Studi Roma Tre, 00146, Roma, Italy; giovanna.desimone@uniroma3.it (G.D.S.); alessandra.dimasi@uniroma3.it (A.d.M.)
2 Dipartimento di Fisica, Università di Genova, 16146, Genova, Italy; pesce@fisica.unige.it
3 Dipartimento di Bioscienze, Università di Milano, 20133, Milano, Italy; martino.bolognesi@unimi.it
4 Centro di Ricerche Pediatriche "R.E. Invernizzi", Università di Milano, 20133, Milano, Italy
5 Dipartimento di Scienze Cliniche e Medicina Traslazionale, Università di Roma "Tor Vergata", 00133, Roma, Italy; chiara.ciaccio@uniroma2.it
6 Dipartimento di Chimica "Ugo Schiff", Università di Firenze, 50019, Sesto Fiorentino (FI), Italy; lorenzo.tognaccini@unifi.it (L.T.); giulietta.smulevich@unifi.it (G.S.)
7 Dipartimento di Scienze Matematiche, Fisiche e Informatiche, Università di Parma, 43124, Parma, Italy; stefania.abbruzzetti@unipr.it (S.A.); cristiano.viappiani@unipr.it (C.V.)
8 Dipartimento di Scienze degli Alimenti e del Farmaco, Università di Parma, 43124, Parma, Italy; stefano.bruno@unipr.it
9 Servizio Grandi Strumentazioni e Core Facilities, Istituto Superiore di Sanità, 00161, Rome, Italy; sara.dellamonaca@iss.it (S.D.M.); paola.fattibene@iss.it (D.P.); donatella.pietraforte@iss.it (P.F.)
10 Laboratorio Interdipartimentale di Microscopia Elettronica, Università Roma Tre, 00146, Roma, Italy

*Correspondence: coletta@seneca.uniroma2.it (M.C.); ascenzi@uniroma3.it (P.A.); Tel.: +39-06-72596365 (M.C.); +39-06-57336363 (P.A.); Fax: +39-06-72596353 (M.C.); +39-06-57336321 (P.A.).

† authors contributed equally to this work.

Table S1. Effect of the CO and NO concentration on values of k_{obs}(CO) and k_{obs}(NO), respectively, for *Mt*-Nb(II) and *Hs*-Nb(II) carbonylation and nitrosylation.

Mt-Nb(II)[a]		*Hs*-Nb(II)[a]	
[CO] (M)	k_{obs}(CO) (s^{-1})	[CO] (M)	k_{obs}(CO) (s^{-1})
2.0×10^{-5}	4.1±0.4 [b]	3.0×10^{-5}	6.4±0.8 [b]
2.3×10^{-5}	4.5±0.5 [b]	4.5×10^{-5}	8.3±0.9 [b]
3.7×10^{-5}	5.7±0.6 [b]	7.0×10^{-5}	10.8±1.4 [b]
5.5×10^{-5}	6.7±0.8 [b]	10.5×10^{-5}	14.4±1.6 [b]
10.0×10^{-5}	8.3±0.8 [b]	21.0×10^{-5}	23.6±2.9 [b]
15.0×10^{-5}	11.4±1.4 [b]		
Mt-Nb(II) [c]		*Hs*-Nb(II) [c]	
[NO] (M)	k_{obs}(NO) (s^{-1})	[NO] (M)	k_{obs}(NO) (s^{-1})
1.5×10^{-5}	27.9±3.1 [d]	1.5×10^{-5}	11.1±1.3 [d]
2.5×10^{-5}	39.2±4.6 [d]	2.5×10^{-5}	26.2±2.5 [d]
4.0×10^{-5}	71.6±6.8 [d]	4.0×10^{-5}	33.9±3.6 [d]
5.0×10^{-5}	88.5±9.3 [d]	5.0×10^{-5}	47.8±5.4 [d]
8.0×10^{-5}	132.7±14.1 [d]	8.0×10^{-5}	72.2±7.1 [d]
10.0×10^{-5}	168.9±18.6 [d]	10.0×10^{-5}	94.8±10.1 [d]

[a] pH 7.0 and 25.0 °C.
[b] [Nb(II))] = 4.0×10^{-6} M.
[c] pH 7.0 and 22.0 °C.
[d] [Nb(II))] = 3.2×10^{-6} M.
